# Awareness of Interventional Radiology Among Medical Students

**DOI:** 10.7759/cureus.69938

**Published:** 2024-09-22

**Authors:** Mohammed Elsayed, Rayan H Mohamed

**Affiliations:** 1 Medicine Department, The James Cook University Hospital, Middlesbrough, GBR; 2 Emergency Department, Bahrain Defence Force Hospital, Riffa, BHR

**Keywords:** interventional radiology, knowledge, medical student, radiology, sudan

## Abstract

Background: Interventional radiology (IR) plays a crucial role in modern medicine, offering minimally invasive treatments for various conditions. Despite its growing significance, the awareness and understanding of IR among medical students are often limited.

Objective: This research aims to evaluate the level of awareness of IR among medical students at the University of Gezira in Sudan

Methodology: This study is a descriptive cross-sectional study that involved 174 medical students from batches 39 and 40. Data was collected through a structured questionnaire designed to assess participants' awareness and perception of IR. The questionnaire was carried out through Google Forms platform, and the data was analyzed using IBM SPSS Statistics for Windows, Version 25 (Released 2017; IBM Corp., Armonk, New York, United States). The chi-square test was used to compare qualitative data, and a p-value of <0.05 was considered significant.

Results: Females were 97 (55.7%) of the participants, while males were 77 (44.3%). The educational level found that 85 (48.9%) are finalists (fifth year) and 89 (51.1%) have graduated. A total of 75 (43.1%) of the medical students considered themselves having poor knowledge about IR. A total of 163 (93%) have confirmed that mandatory radiology course is important to be added to the curriculum of the medical school. Furthermore, 149 (85.6%) are familiar with the concept of angioplasty, and 60 (34.5%) have seen patients treated by an interventional radiologist. Most of the students are not considering IR as a career mainly due to the lack of knowledge or fear from radiological exposure.

Conclusion: Medical students’ awareness and perception of IR is inadequate which may adversely affect their consideration of pursuing it as a career path. Educational interventions aimed at enhancing medical students' knowledge and interest in IR may be needed to improve patient outcomes and tackle healthcare challenges.

## Introduction

Diagnostic radiology encompasses several subspecialties, including neuroradiology, paediatric radiology, nuclear radiology, hospice and palliative care, pain management, and vascular and interventional radiology (IR) [1]. Among these, IR stands out as a dynamic and rapidly evolving field. It utilizes imaging technologies such as X-rays, computed tomography (CT) scans, magnetic resonance imaging (MRI), and ultrasound to guide minimally invasive diagnostic and therapeutic procedures [2]. 

IR includes a broad spectrum of procedures designed to treat various medical conditions. These procedures include central venous catheterization, embolization of arteries and veins to control haemorrhage, image-guided biopsy, percutaneous drainage, drain implantation, and image-guided tumour ablation and chemotherapy [3]. The ability to perform such procedures using minimally invasive techniques offers significant benefits, such as reduced patient recovery time and minimized procedural risks, making IR an integral part of modern medical practice.

Previous studies have consistently shown that medical students, in particular, have limited knowledge and awareness of IR [4-7]. Despite some medical schools in the US exposing students to IR, only 5.5% participated in elective rotations, and among those students, only 12.7% were interested in IR as a specialty [7]. The level of awareness and understanding of IR remains rather limited overall, particularly among students in their preclinical years due to the absence of radiology rotations until the clinical years [5,6,8,9].

In Saudi Arabia, a study conducted by Meshari et al. [10] emphasized the need for increased awareness of IR among medical students to improve patient outcomes and address healthcare challenges. This need is similarly critical in Sudan, where there are currently only four interventional radiologists, all of whom received their training abroad due to the lack of local training programs [11]. As medical services in Sudan continue to develop, there is an urgent need to integrate IR into the medical curriculum to prepare future healthcare professionals adequately.

This study aims to evaluate the awareness and perception of IR among medical students at the University of Gezira, Sudan. By assessing the current level of knowledge and understanding, the research seeks to identify gaps in the curriculum and suggest improvements. Enhancing IR education among medical students is expected to foster better integration of these techniques into clinical practice, ultimately improving patient care outcomes in the region. Additionally, the findings of this study could provide valuable insights for other medical schools in similar settings, highlighting the importance of incorporating IR into medical training programs to meet the evolving needs of modern healthcare.

## Materials and methods

Study design and setting

This study is a descriptive cross-sectional study conducted at the University of Gezira, Sudan, after obtaining approval from its Research Ethics Committee (approval number: 1/2024) with informed consent from all participants. This study was conducted over a period of three months from March 2024 to June 2024. The study aimed to assess the knowledge of medical students about IR.

Study population

The participants were from two batches of medicine, batch 39 who have recently graduated and batch 40 who are on their final year as medical students at the University of Gezira. Students who refused to participate in the study and those who were in junior years were excluded.

Sample size

Sample size was calculated based on the total number of medical students (550), with a confidence interval of 95% and a margin of error of 6.15%, which revealed 174 medical students.

Data collection tool

Data was collected through a structured questionnaire (see Appendix, Table 8) adapted from a previous study conducted in Saudi Arabia by Shafiq et al. [12]. There were 18 questions in the survey. The questionnaire included questions about their demographic data such as gender and batch number and questions aimed to assess interest in, degree of knowledge about, and willingness to learn more about IR. The questionnaire was carried out through Google Forms platform.

Data analysis

Data collected from the questionnaires were analyzed using IBM SPSS Statistics for Windows, Version 25 (Released 2017; IBM Corp., Armonk, New York, United States). Qualitative variables were presented in the form of frequency and percentages. We used the chi-square analysis to compare the proportions of qualitative variables between the two groups. A p-value of less than 0.05 was considered significant. 

## Results

Table 1 shows the demographic distribution of the study population. The sample consists of a slightly higher proportion of females, who make up 97 (55.7%) of the respondents, compared to males, who represent 77 (44.3%). In terms of educational level, the distribution is nearly even, with 85 (48.9%) of the respondents being fifth-year students from batch 40, while 89 (51.1%) are recent graduates from batch 39. This balanced representation across gender and educational level suggests that the questionnaire results are likely to capture the perspectives of both current students and recent graduates, with a slight majority being female and recently graduated individuals.

**Table 1:** The descriptive statistics for the gender and educational level of the study population (n = 174)

Variable		Frequency	Percentage
Gender	Female	97	55.7%
	Male	77	44.3%
Level	5th year (batch 40)	85	48.9%
	Graduated (batch 39)	89	51.1%

The data in Table 2 revealed that while respondents generally felt somewhat confident in their knowledge of radiology, with 81 (46.6%) rating it as 'Adequate', their understanding of IR was notably weaker, with 75 (43.1%) rating their knowledge as 'Poor', and none rating it as 'Excellent'. Regarding career prospects in IR, opinions were mixed; 60 (34.5%) viewed them as 'Good', but 38 (21.8%) were uncertain. Out of the respondents, 109 (62.6%) were not considering careers in diagnostic radiology, and 107 (61.5%) were not inclined towards IR, although a notable minority remained open to these career paths. A total of 163 respondents (93.7%) supported the establishment of a mandatory radiology course in medical school, highlighting the perceived need for better education in this area. While many respondents identified that interventional radiologists were involved in outpatient clinics, ward rounds, and patient admissions, there was still some uncertainty, suggesting gaps in their understanding of the specialty's full scope.

**Table 2:** General perception of the medical students about radiology (n = 174)

Variable		N	%
How do you rate your knowledge of radiology in general compared to other subjects?	No knowledge	2	1.1%
	Poor	51	29.3%
	Adequate	81	46.6%
	Good	39	22.4%
	Excellent	1	.6%
How do you rate your knowledge of interventional radiology in general compared to other subjects?	No knowledge	20	11.5%
	Poor	75	43.1%
	Adequate	49	28.1%
	Good	30	17.2%
	Excellent	0	0%
What do you think about the career prospects for interventional radiology?	Don’t know	38	21.8%
	Poor	11	6.3%
	Adequate	28	16.1%
	Good	60	34.5%
	Excellent	37	21.3%
Would you consider a career in diagnostic radiology?	No	109	62.6%
	Not sure	15	8.6%
	Yes	50	28.7%
Would you consider a career in interventional radiology?	No	107	61.5%
	Not sure	13	7.5%
	Yes	54	31.0%
Do you think a mandatory radiology course during medical school would be beneficial?	No	2	1.1%
	Not sure	9	5.2%
	Yes	163	93.7%
Interventional radiologists have outpatient clinics	True	102	58.6%
	False	72	41.4%
Interventional radiologists do ward round in the hospital	True	103	59.2%
	False	71	40.8%
Interventional radiologists admit patients to the hospital	True	111	63.8%
	False	63	36.2%

The data in Table 3 indicated that a substantial majority of respondents believe that interventional radiologists perform various procedures. Specifically, 119 (68.4%) thought they carry out cardiac angioplasty or stenting, 100 (57.5%) believed they perform femoral-popliteal arterial bypass, 107 (61.5%) acknowledged venous access procedures, 102 (58.6%) supported arteriovenous fistulas for dialysis, and 109 (62.6%) recognized lower limb angioplasty and stenting. In contrast, 67 respondents (38.5%) considered that interventional radiologists perform uterine artery embolization for fibroids.

**Table 3:** Perception of medical students in general about interventional radiology (n = 174)

Variable	Yes		No	
	N	%	N	%
An interventional radiologist performs the following procedures:				
Cardiac angioplasty or stenting	119	68.4%	55	31.6%
Femoral-popliteal arterial bypass	100	57.5%	74	42.5%
Venous access procedures (e.g., Hickman line)	107	61.5%	67	38.5%
Arteriovenous fistulas for dialysis	102	58.6%	72	41.4%
Uterine artery embolization for fibroids	67	38.5%	107	61.5%
Lower limb angioplasty and stenting	109	62.6%	65	37.4%
Is any of the following procedures routinely performed by IR?				
Vertebroplasty	81	46.6%	93	53.4%
Radiofrequency ablation of tumours	99	56.9%	75	43.1%
Endovascular repair of aortic aneurysms	107	61.5%	67	38.5%
Percutaneous nephrostomy	76	43.7%	98	56.3%
Image-guided tumour biopsy	115	66.1%	59	33.9%
Are you familiar with the procedure called ‘angioplasty’	149	85.6%	25	14.4%
If yes, from where				
Cardiologist	113	64.9%	32	18.4%
Vascular surgeon	122	70.1%	22	12.6%
Interventional radiologist	54	31.0%	87	50.0%
Others	58	33.3%	80	46.0%
Have you seen patients who were treated by an interventional radiologist?	60	34.5%	114	65.5%
An interventional radiologist must complete training in:				
Radiology	155	89.1%	19	10.9%
Surgery	97	55.7%	77	44.3%
Both radiology and surgery	81	46.6%	93	53.4%
Internal medicine	46	26.4%	128	73.6%

Regarding routinely performed procedures, 81 (46.6%) identify vertebroplasty, 99 (56.9%) radiofrequency ablation of tumours, 107 (61.5%) endovascular repair of aortic aneurysms, and 115 (66.1%) image-guided tumour biopsy, while 76 (43.7%) acknowledge percutaneous nephrostomy. A large majority, 149 (85.6%), are familiar with angioplasty, with the majority learning about it from cardiologists (113, 64.9%), vascular surgeons (122, 70.1%), or interventional radiologists (54, 31.0%). Conversely, 60 (34.5%) have seen patients treated by interventional radiologists. Most respondents believe that interventional radiologists should complete training in radiology (155, 89.1%), while fewer support training in surgery (97, 55.7%), both radiology and surgery (81, 46.6%), or internal medicine (46, 26.4%).

**Figure 1:**
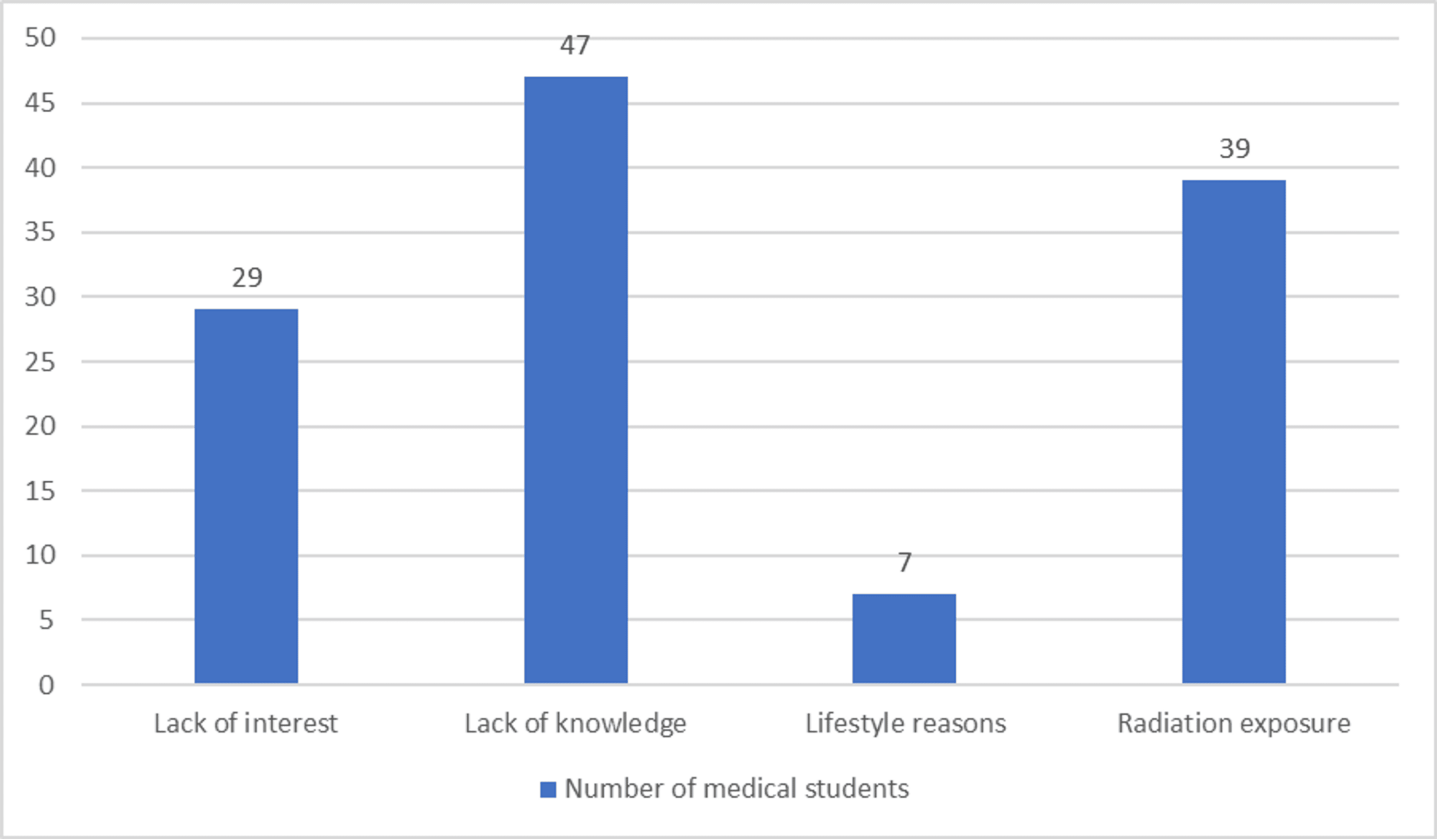
Reasons why medical students are not considering interventional radiology as a career X-axis: Number of medical students; Y-axis: reasons for not considering IR as a career A total of 47 participants cited lack of knowledge as the reason for not considering interventional radiology as a career option. Additionally, 39 participants attributed their decision to concerns about radiation exposure, while 29 indicated a lack of interest in the field. Only seven students identified lifestyle factors as a determining reason

In Figure 1, most of the participants are not considering IR as a career mainly due to the lack of knowledge (47).

A notable gender difference appeared in the support for a mandatory radiology course during medical school, with female respondents supporting it compared to males (Chi-square = 7.54, p = 0.023). Also, another difference was noted in perceptions of hospital admissions by interventional radiologists with more females recognizing this role compared to males (Chi-square = 6.65, p = 0.010). In general, there were no significant gender differences in ratings of radiology knowledge (Chi-square = 5.64, p = 0.228) or IR knowledge (Chi-square = 4.22, p = 0.239). Career prospects for IR also showed no significant gender differences (Chi-square = 7.12, p = 0.130). Both male and female respondents largely rejected careers in diagnostic radiology and IR, with no significant gender differences (Chi-square = 1.451, p = 0.484 and Chi-square = 2, p = 0.368). No significant differences were observed between male and female participants regarding their awareness of specific characteristics of interventional radiologists, such as whether these professionals have outpatient clinics (p = 0.331) or conduct hospital ward rounds (p = 0.155) as shown in Table 4.

**Table 4:** The correlation between gender and aspects of general perception about radiology p-value is considered significant if p < 0.05. ^*^Significant correlation

Variable		Gender				Total	Chi-square, p-value
		Female		Male			
		N	%	N	%		
How do you rate your knowledge of radiology in general compared to other subjects?	Adequate	50	28.7%	31	17.8%	81 (46.6%)	5.64, 0.228
	Good	18	10.3%	21	12.1%	39 (22.4%)	
	No knowledge	2	1.1%	0	0%	2 (1.1%)	
	Poor	27	15.5%	24	13.8%	51 (29.3%)	
	Excellent	0	0%	1	0.57%	1 (0.57%)	
How do you rate your knowledge of interventional radiology in general compared to other subjects?	Adequate	31	18.8%	18	10.3%	49 (28.1%)	4.22, 0.239
	Good	12	6.8%	18	10.3%	30 (17.2%)	
	No knowledge	11	6.3%	9	5.2%	20 (11.5%)	
	Poor	43	24.7%	32	18.4%	75 (43.1%)	
	Excellent	0	0%	0	0%	0	
What do you think about the career prospects for interventional radiology?	Adequate	16	9.2%	12	6.7%	28 (16.1%)	7.12, 0.130
	Good	33	18.9%	27	15.5%	60 (34.4%)	
	Don’t know	27	15.5%	11	6.3%	38 (21.8%)	
	Poor	6	3.4%	5	2.8%	11 (6.3%)	
	Excellent	15	8.6%	22	12.6%	37 (21.3%)	
Would you consider a career in diagnostic radiology?	No	62	35.6%	47	22.0%	109 (62.6%)	1.451, 0.484
	Not sure	10	5.7%	5	2.9%	15 (8.6%)	
	Yes	25	14.4%	25	14.4%	50 (28.8)	
Would you consider a career in diagnostic interventional radiology?	No	64	36.8%	43	24.7%	107 (61.5%)	2, 0.368
	Not sure	7	4.0%	6	3.5%	13 (7.5%)	
	Yes	26	14.9%	28	16.1%	54 (31.0%)	
Do you think a mandatory radiology course during medical school would be beneficial?	No	1	0.6%	1	0.6%	2 (1.1)	7.54, *0.023
	Not sure	9	5.1%	0	0%	9 (5.2)	
	Yes	87	50.0%	76	43.7%	163 (93.7%)	
Interventional radiologists have outpatient clinics.	False	37	21.3%	35	20.1%	72 (41.4%)	0.946, 0.331
	True	60	34.5%	42	24.1%	102 (58.6%)	
Interventional radiologists do ward rounds in the hospital.	False	35	20.1%	36	20.7%	71 (40.8%)	2.024, 0.155
	True	62	35.6%	41	23.6%	103 (59.2%)	
Interventional radiologists admit patients to the hospital.	False	27	15.5%	36	20.7%	63 (36.2%)	6.65, *0.010
	True	70	40.3%	41	23.6%	111 (63.8%)	

The analysis of responses between female and male participants revealed no significant differences in their perception of various procedures being done by interventional radiologists. For example, the recognition of cardiac angioplasty or stenting (Chi-square = 0.012, p = 0.911), femoral-popliteal arterial bypass (Chi-square = 0.719, p = 0.396), and venous access procedures (Chi-square = 0.012, p = 0.912) was similar between genders. The perception of arteriovenous fistulas (Chi-square = 1.433, p = 0.231), uterine artery embolization (Chi-square = 0.18, p = 0.672), and lower limb angioplasty (Chi-square = 0.761, p = 0.383) also did not show significant variance. Routine IR procedures like vertebroplasty (Chi-square = 0.319, p = 0.572), radiofrequency ablation (Chi-square = 0.311, p = 0.577), endovascular repair of aortic aneurysms (Chi-square = 0.042, p = 0.839), and percutaneous nephrostomy (Chi-square = 0.038, p = 0.846) were similarly recognized by both genders. Familiarity with angioplasty (Chi-square = 0.214, p = 0.644) and exposure to patients treated by IR specialists (Chi-square = 0.216, p = 0.642) did not differ significantly between male and female respondents. Furthermore, perceptions regarding the required training for IR specialists in radiology (Chi-square = 0.399, p = 0.842), surgery (Chi-square = 0.719, p = 0.396), both fields (Chi-square = 0.435, p = 0.510), or internal medicine (Chi-square = 1.349, p = 0.245) were consistent across genders as seen in Table 5.

**Table 5:** The correlation between gender and aspects of specific perception about radiology p-value is considered significant if p < 0.05

Variable		Gender				Total	Chi-square, p-value
		Female		Male			
		N	%	N	%		
An interventional radiologist performs the following procedures:							
Cardiac angioplasty or stenting	No	31	17.8%	24	13.8%	55 (31.6%)	0.012, 0.911
	Yes	66	37.9%	53	30.5%	119 (68.4%)	
Femoral-popliteal arterial bypass	No	44	25.3%	30	17.2%	74 (42.5%)	0.719, 0.396
	Yes	53	30.5%	47	27.01%	100 (57.5%)	
Venous access procedures (e.g., Hickman line)	No	37	21.3%	30	17.2%	67 (38.5%)	0.012, 0.912
	Yes	60	34.5%	47	21.0%	107 (61.5%)	
Arteriovenous fistulas for dialysis	No	44	25.3%	28	16.1%	72 (41.4%)	1.433, 0.231
	Yes	53	30.5%	49	28.16%	102 (58.6%)	
Uterine artery embolization for fibroids	No	61	35.1%	46	26.43%	107 (61.5%)	0.18, 0.672
	Yes	36	20.7%	31	17.8%	67 (38.5%)	
Lower limb angioplasty and stenting	No	39	22.4%	26	14.9%	65 (37.4%)	0.761, 0.383
	Yes	58	33.3%	51	29.3%	109 (62.6%)	
Is any of the following procedures routinely performed by IR?							
Vertebroplasty	No	50	28.7%	43	24.7%	93 (53.4%)	0.319, 0.572
	Yes	47	27.0%	34	19.5%	81 (46.6%)	
Radiofrequency ablation of tumours	No	40	22.9%	35	20.1%	75 (43.1%)	0.311, 0.577
	Yes	57	32.8%	42	24.13%	99 (56.9%)	
Endovascular repair of aortic aneurysms	No	38	21.8%	29	16.7%	67 (38.5%)	0.042, 0.839
	Yes	59	33.9%	48	27.6%	107 (61.5%)	
Percutaneous nephrostomy	No	54	31.0%	44	25.3%	98 (56.3%)	0.038, 0.846
	Yes	43	24.7%	33	18.9%	76 (43.7%)	
Image-guided tumour biopsy	No	35	20.1%	24	13.8%	59 (33.9%)	0.463, 0.497
	Yes	62	35.6%	53	30.5%	115 (66.1%)	
Are you familiar with the procedure called ‘angioplasty’	No	15	8.6%	10	5.7%	25 (14.5%)	0.214, 0.644
	Yes	82	47.1%	67	38.5%	149 (85.6%)	
Have you seen patients who were treated by an interventional radiologist?	No	65	37.4%	49	28.2%	114 (65.5%)	0.216, 0.642
	Yes	32	18.4%	28	16.1%	60 (34.5%)	
An interventional radiologist must complete training in							
Radiology	No	11	6.3%	8	4.6%	19 (10.9%)	0.399, 0.842
	Yes	86	49.4%	69	39.7%	155 (89.1%)	
Surgery	No	44	25.3%	33	18.9%	77 (44.3%)	0.719, 0.396
	Yes	53	30.5%	44	25.3%	97 (55.7%)	
Both radiology and surgery	No	54	31.0%	39	22.4%	93 (53.4%)	0.435, 0.51
	Yes	43	24.7%	38	21.8%	81 (46.6%)	
Internal medicine	No	68	39.1%	60	34.5%	128 (73.6%)	1.349, 0.245
	Yes	29	16.7%	17	9.8%	46 (26.4%)	

The comparison between fifth-year students and recent graduates revealed no significant differences in their self-assessed knowledge of radiology and IR, with both groups generally rating their knowledge as 'adequate' or 'poor' (Chi-square = 7.44, p = 0.114 for radiology knowledge; Chi-square = 5.38, p = 0.148 for IR knowledge). Career prospects in IR were viewed similarly across both groups, with no significant differences in their responses (Chi-square = 5.62, p = 0.230). Most respondents, regardless of educational level, were hesitant to pursue careers in diagnostic radiology (Chi-square = 1.46, p = 0.482) or IR (Chi-square = 0.661, p = 0.718), and there was broad consensus on the need for a mandatory radiology course during medical school (Chi-square = 4.743, p = 0.093). Additionally, both groups had similar perceptions regarding the clinical roles of interventional radiologists, including outpatient clinics (Chi-square = 0.954, p = 0.329), ward rounds (Chi-square = 0.01, p = 0.922), and hospital admissions (Chi-square = 1.42, p = 0.233) as shown in Table 6.

**Table 6:** The correlation between the level with aspects of general perception of radiology p-value is considered significant if p < 0.05

Variable		Level				Total	Chi-square, p-value
		5th year (batch 40)		Graduated (batch 39)			
		N	%	N	%		
How do you rate your knowledge of radiology in general compared to other subjects?	Adequate	46	26.4%	35	20.1%	81 (46.6%)	7.44, 0.114
	Excellent	0	0%	1	0.57%	1 (0.57%)	
	Good	15	8.6%	24	13.8%	39 (22.4%)	
	No knowledge	2	1.1%	0	0%	2 (1.1%)	
	Poor	22	12.6%	29	16.67%	51 (29.3%)	
How do you rate your knowledge of interventional radiology in general compared to other subjects?	Adequate	26	14.9%	23	14.2%	49 (28.1%)	5.38, 0.148
	Excellent	0	0%	0	0%	0	
	Good	10	5.7%	20	11.5%	30 (17.2%)	
	No knowledge	13	7.5%	7	4.0%	20 (11.5%)	
	Poor	36	20.7	39	22.4%	75 (43.1%)	
What do you think about the career prospects for Interventional Radiology?	Adequate	14	8.0%	14	8.0%	28 (16.1%)	5.62, 0.230
	Don't know	24	13.8%	14	8.0%	38 (21.8%)	
	Excellent	14	8.0%	23	13.2%	37 (21.3%)	
	Good	29	16.7%	31	17.8%	60 (34.5%)	
	Poor	4	2.3%	7	4.0%	11 (6.3%)	
Would you consider a career in diagnostic radiology?	No	57	32.8%	52	29.9%	109	1.46, 0.482
	Not sure	6	3.4%	9	5.2%	15	
	Yes	22	12.6%	28	16.1%	50	
Would you consider a career in interventional radiology?	No	54	31.0%	53	30.5%	107	0.661, 0.718
	Not sure	7	4.0%	6	3.5%	13	
	yes	24	13.8%	30	17.2%	54	
Do you think a mandatory radiology course during medical school would be beneficial?	No	0	0%	2	1.1%	2 (1.1%)	4.743, 0.093
	Not sure	2	1.1%	7	4.0%	9 (5.2%)	
	Yes	83	47.7%	80	45.9%	163 (93.7%)	
Interventional radiologists have outpatient clinics.	False	32	18.4%	40	22.9%	72 (41.4%)	0.954, 0.329
	True	53	30.5%	49	28.1%	102 (58.6%)	
Interventional radiologists do ward rounds in the hospital.	False	35	20.1%	36	20.7%	71 (40.8%)	0.01, 0.922
	True	50	28.7%	53	30.5%	103 (59.2%)	
Interventional radiologists admit patients to the hospital.	False	27	15.5%	36	20.7%	63 (36.2%)	1.42, 0.233
	True	58	33.3%	53	30.5%	111 (63.8%)	

The data in Table 7 revealed that there were no significant differences in the perception of procedures between fifth-year students and graduates. For instance, recognition of cardiac angioplasty or stenting (Chi-square = 1.044, p = 0.307), femoral-popliteal arterial bypass (Chi-square = 0.322, p = 0.570), and venous access procedures (Chi-square = 0.007, p = 0.933) was similar across both groups. Awareness of other IR procedures such as arteriovenous fistulas (Chi-square = 1.651, p = 0.199), uterine artery embolization (Chi-square = 0.291, p = 0.590), and lower limb angioplasty (Chi-square = 0.496, p = 0.480) also showed no significant variation. Knowledge of routinely performed IR procedures, including vertebroplasty (Chi-square = 0.610, p = 0.435), radiofrequency ablation (Chi-square = 0.252, p = 0.616), and endovascular repair of aortic aneurysms (Chi-square = 0.157, p = 0.692), did not differ significantly between the two educational levels. However, familiarity with angioplasty showed a significant difference (Chi-square = 4.285, p = 0.0386), indicating a notable gap. There were no significant differences in familiarity with IR procedures or perceptions regarding required training in radiology, surgery, both, or internal medicine (Chi-square values ranged from 0.03 to 2.703, p-values from 0.1 to 0.862).

**Table 7:** The correlation between the level of education and aspects of specific perception of radiology p-value is considered significant if p < 0.05. ^*^Significant correlation

Variable			Level of education				Total	Chi-square, p-value	
			5th year (batch 40)		Graduated (batch 39)				
			N	%	N	%			
An interventional radiologist performs the following procedures:									
Cardiac angioplasty or stenting	No	30		17.2%	25	14.4%	55 (31.6%)		1.044, 0.307
	Yes	55		31.6%	64	36.8%	119 (68.4%)		
Femoral-popliteal arterial bypass	No	38		21.9%	36	20.7%	74 (42.5%)		0.322, 0.57
	Yes	47		20.01%	53	30.5%	100 (57.5%)		
Venous access procedures (e.g., Hickman line)	No	33		18.9%	34	19.5%	67 (38.5%)		0.007, 0.933
	Yes	52		29.9%	55	31.6%	107 (61.5%)		
Arteriovenous fistulas for dialysis	No	31		17.8%	41	23.6%	72 (41.4%)		1.651, 0.199
	Yes	54		31.0%	48	27.6%	102 (58.6%)		
Uterine artery embolization for fibroids	No	54		31.0%	53	30.5%	107 (61.5%)		0.291, 0.59
	Yes	31		17.8%	36	20.7%	67 (38.5%)		
Lower limb angioplasty and stenting	No	34		19.5%	31	17.8%	65 (37.4%)		0.496, 0.48
	Yes	51		29.3%	58	33.3%	109 (62.6%)		
Is any of the following procedures routinely performed by IR?									
Vertebroplasty	No	48		27.6%	45	25.9%	93 (53.4%)		0.61, 0.435
	Yes	37		21.3%	44	25.3%	81 (46.6%)		
Radiofrequency ablation of tumours	No	35		20.1%	40	22.9%	75 (43.1%)		0.252, 0.616
	Yes	50		28.7%	49	28.2%	99 (56.9%)		
Endovascular repair of aortic aneurysms	No	34		19.5%	33	18.9%	67 (38.5%)		0.157, 0.692
	Yes	51		29.3%	56	32.1%	107 (61.5%)		
Percutaneous nephrostomy	No	48		27.6%	50	28.7%	98 (56.3%)		0.0015, 0.969
	Yes	37		21.3%	39	22.4%	76 (43.7%)		
Image-guided tumour biopsy	No	31		17.8%	28	16.1%	59 (33.9%)		0.487, 0.485
	Yes	54		31.0%	61	35.1%	115 (66.1%)		
Are you familiar with the procedure called ‘angioplasty’	No	17		9.8%	8	4.6%	25 (14.5%)		4.285, 0.0386*
	Yes	68		39.1%	81	46.6%	149 (85.6%)		
Have you seen patients who were treated by an interventional radiologist?	No	59		33.9%	55	31.6%	114 (65.5%)		1.116, 0.291
	Yes	26		14.9%	34	19.5%	60 (34.9%)		
An interventional radiologist must complete training in									
Radiology	No		6	3.5%	13	7.5%	19 (10.9%)		2.546, 0.11
	Yes		79	45.4%	76	43.7%	155 (89.1%)		
Surgery	No		43	24.7%	34	19.5%	77 (44.3%)		2.703, 0.1
	Yes		42	24.1%	55	31.6%	97 (55.7%)		
Both radiology and surgery	No		46	31.1%	47	22.4%	93 (53.4%)		0.03, 0.862
	Yes		39	24.7%	42	21.8%	81 (46.6%)		
Internal medicine	No		62	35.6%	66	37.9%	128 (73.6%)		0.033, 0.856
	Yes		23	13.2%	23	13.2%	46 (26.4%)		

## Discussion

IR is a rapidly growing field that faces several challenges including lack of awareness and shortage of manpower [13]. Medical students are crucial to the growth of IR as they are the potential future interventional radiologists and referring doctors [5]. Their education and exposure to IR during medical training are essential in shaping the next generation of practitioners who will drive innovation and improve patient care through minimally invasive techniques.

Radiology was previously not educated in Sudanese medical schools as a separate discipline. Currently, the medical schools started to teach it separately and give more details about the field and subspecialties. As far as we are aware, there is no literature on medical students’ knowledge about IR as a specialty in Sudan. This lack of knowledge could be keeping them from concentrating on IR and possibly contributing to the perception that IR is a minor specialty. These misunderstandings could significantly influence the decision to pursue radiology or IR as a career.

IR is inadequately integrated into the undergraduate medical curriculum. In this study, 163 (93.7%) of participants believed that making radiology a mandatory course in medical school would be beneficial, despite not all of them planning to specialize in radiology after graduation. Medical students who had a required radiology rotation would likely possess greater knowledge about IR as a specialty compared to those who did not have such a rotation [9].

This study revealed that among the participants, 97 (55.7%) were females and 77 (44.3%) were males. Self-reported knowledge of IR showed that 20 (11.5%) had 'no knowledge', 75 (43.1%) had 'poor knowledge', 49 (28.2%) had 'adequate knowledge', 30 (17.2%) had 'good knowledge', and no participant had 'excellent knowledge', demonstrating poor awareness of this field.

Comparable results were noted in a study conducted in Saudi Arabia in which 52% of students exhibited poor knowledge of IR [5]. These results are in line with research from Ireland, where 62% of medical students had inadequate knowledge of IR [14], and studies from England [8] and Canada [6], where 52% and 55.5% of students, respectively, had little knowledge of IR as a specialty. Only 54 (31%) of the participants had considered IR as a career. The most commonly cited reason for either not choosing or being uncertain about a career in IR was a lack of knowledge in the field, with 47 participants (38.5%) indicating this. The same finding was documented by Sara et al [1], and this should lead to increased efforts to address this knowledge gap by raising awareness and exposing students to the specialty.

Notably, 155 (89.1%) of respondents correctly identified the IR training path; however, a significant gap in knowledge about the clinical practices of interventional radiologists was evident, as 119 (68.4%) and 100 (57.5%) of respondents mistakenly believed that interventional radiologists performed cardiac angioplasty and femoral-popliteal bypass, respectively. Additionally, the majority of responders were unfamiliar with various IR procedures such as vertebroplasty and percutaneous nephrostomy. One-third or more of the respondents did not think interventional radiologists admit patients, do ward rounds, or have outpatient clinics, a similar result was found by Alshumrani [5]. Lack of exposure and understanding as factors contributing to low awareness of IR as a specialty have also been covered in other researches [6,9,15].

In this study, we found that females were significantly more interested in having mandatory radiology course during medical school (p-value = 0.023). Similarly, females were significantly more agreeing that interventional radiologists admit patients to the hospital than their counterparts (p-value = 0.010). In addition, graduated students were significantly more familiar with the procedure 'Angioplasty' than final-year medical students with a (p-value = 0.039).

This study has certain limitations. The findings are specific to a single Sudanese university and are based on a relatively small sample size. It would be necessary to conduct larger national studies involving several Sudanese universities in order to get precise results. Self-reporting bias may have resulted from participants giving answers based on perceived expectations or social desirability than on their actual knowledge and interest in IR.

## Conclusions

The study highlights a significant gap in medical students' awareness and perception of IR among medical students at Gezira University. This deficiency in understanding is concerning, as it may lead to a reduced interest in IR as a specialty, despite its critical role in modern medicine. This gap not only impacts students' career decisions but also has broader implications for the future of IR as a field, given the increasing demand for skilled interventional radiologists. Therefore, there is a pressing need to implement targeted educational strategies within medical training programs to enhance students' understanding of IR. Such initiatives should aim to provide detailed insights into the scope and impact of IR procedures, thereby enabling students to make more informed career choices and potentially increasing the number of specialists in this vital area of medicine.

## Appendices

**Table 8:** Questionnaire The questionnaire used in this study was adapted from Shafiq et al. [13], Level of Awareness Regarding Interventional Radiology Among Medical Students at Northern Border University in Arar, Saudi Arabia, with permission from the authors. We gratefully acknowledge the authors for granting us permission to use and adapt their work in our research

Question	Answer
1- Gender	❑ male ❑ female
2- What is your level?	❑ 5th year (batch 40) ❑ Graduated (batch 39)
3- How do you rate your knowledge of radiology in general compared to other subjects?	a. Excellent b. Good c. Adequate d. Poor e. No knowledge
4- How do you rate your knowledge of interventional radiology in general compared to other subjects?	a. Excellent b. Good c. Adequate d. Poor e. No knowledge
5- An interventional radiologist performs the following procedures:	
a. Cardiac angioplasty or stenting	❑ Yes ❑ No
b. Femoral-popliteal arterial bypass	❑ Yes ❑ No
c. Venous access procedures (e.g., Hickman line)	❑ Yes ❑ No
d. Arteriovenous fistulas for dialysis	❑ Yes ❑ No
e. Uterine artery embolization for fibroids	❑ Yes ❑ No
f. Lower limb angioplasty and stenting	❑ Yes ❑ No
6- Is any of the following procedures routinely performed by IR?	
a. Vertebroplasty	❑ Yes ❑ No
b. Radiofrequency ablation of tumours	❑ Yes ❑ No
c. Endovascular repair of aortic aneurysms	❑ Yes ❑ No
d. Percutaneous nephrostomy	❑ Yes ❑ No
e. Image-guided tumour biopsy	❑ Yes ❑ No
7-Are you familiar with the procedure called ‘angioplasty’?	❑ Yes ❑ No
8- If yes, did you gain that exposure through:	
a. Cardiologist	❑ Yes ❑ No
b. Vascular surgeon	❑ Yes ❑ No
c. Interventional radiologist	❑ Yes ❑ No
d. Others	❑ Yes ❑ No
9- Would you consider a career in diagnostic radiology?	❑ Yes ❑ No ❑ Not sure
10- Would you consider a career in interventional radiology?	❑ Yes ❑ No ❑ Not sure
11- If you answered No or Not sure to the previous question, please choose the most appropriate reason why.	❑ Lack of knowledge ❑ Lack of interest ❑ Lifestyle reasons ❑ Radiation exposure
12- Have you seen patients who were treated by an interventional radiologist?	❑ Yes ❑ No
13- An interventional radiologist must complete training in:	a. Radiology b. Surgery c. Both radiology and surgery d. Internal medicine
14- Interventional radiologists have outpatient clinics.	❑ True ❑ False
15- Interventional radiologists do ward rounds in the hospital.	❑ True ❑ False
16- Interventional radiologists admit patients to the hospital	❑ True ❑ False
17- Do you think a mandatory radiology course during medical school would be beneficial?	❑ Yes ❑ No ❑ Not sure
18- What do you think about the career prospects for interventional radiology?	a. Excellent b. Good c. Adequate d. Poor e. Don't know
